# Comparative Analysis of the Tyr-Kinases CapB1 and CapB2 Fused to Their Cognate Modulators CapA1 and CapA2 from *Staphylococcus aureus*

**DOI:** 10.1371/journal.pone.0075958

**Published:** 2013-10-11

**Authors:** Jakub Gruszczyk, Vanesa Olivares-Illana, Julien Nourikyan, Aurore Fleurie, Emmanuelle Béchet, Virginie Gueguen-Chaignon, Céline Freton, Magali Aumont-Nicaise, Solange Moréra, Christophe Grangeasse, Sylvie Nessler

**Affiliations:** 1 Laboratoire d’Enzymologie et Biochimie Structurales (LEBS), Centre National de la Recherche Scientifique (CNRS), Gif sur Yvette, France; 2 Institut de Biologie et Chimie des Protéines (IBCP), CNRS/Université Claude Bernard Lyon 1 (UCBL), Lyon, France; 3 Institut de Biochimie et Biophysique Moléculaire et Cellulaire (IBBMC), Université Paris-Sud/CNRS, Orsay, France; Instituto de Tecnologica Química e Biológica, UNL, Portugal

## Abstract

A particular class of tyrosine-kinases sharing no structural similarity with eukaryotic tyrosine-kinases has been evidenced in a large array of bacterial species. These bacterial tyrosine-kinases are able to autophosphorylate on a C-terminal tyrosine-rich motif. Their autophosphorylation has been shown to play a crucial role in the biosynthesis or export of capsular polysaccharide. The analysis of the first crystal structure of the staphylococcal tyrosine kinase CapB2 associated with the activating domain of the transmembrane modulator CapA1 had brought conclusive explanation for both the autophosphorylation and activation processes. In order to explain why CapA1 activates CapB2 more efficiently than its cognate transmembrane modulator CapA2, we solved the crystal structure of CapA2B2 and compared it with the previously published structure of CapA1B2. This structural analysis did not provide the expected clues about the activation discrepancy observed between the two modulators. *Staphylococcus aureus* also encodes for a CapB2 homologue named CapB1 displaying more than 70% sequence similarity and being surprisingly nearly unable to autophosphorylate. We solved the crystal structure of CapA1B1 and carefully compare it with the structure of CapA1B2. The active sites of both proteins are highly conserved and the biochemical characterization of mutant proteins engineered to test the importance of small structural discrepancies identified between the two structures did not explain the inactivity of CapB1. We thus tested if CapB1 could phosphorylate other protein substrates or hydrolyze ATP. However, no activity could be detected in our *in vitro* assays. Taken together, these data question about the biological role of the homologous protein pairs CapA1/CapB1 and CapA2/CapB2 and we discuss about several possible interpretations.

## Introduction

A new family of protein tyrosine-kinases harboring a P-loop type nucleotide binding-domain [1] with a Walker A motif [2] has been described. They have been called BY-kinases (Bacterial-tyrosine-kinases) as they are only found in bacterial genomes [3], [4]. They have been proposed to participate in the regulation of several cellular processes but to date, their best-documented cellular function concerns capsular polysaccharide synthesis in which they act as co-polymerase in the multiprotein capsule assembly machinery [5]. BY-kinase autophosphorylation on a C-terminal tyrosine cluster, called Y-cluster [6] is crucial in regulating polysaccharide export. BY-kinases are also able to phosphorylate various endogenous substrates [7] among which proteins involved in polysaccharide precursor synthesis [8], [9]. For example, in *S. aureus*, the BY-kinase CapB2 has been shown to phosphorylate the UDP-N-acetyl-mannosamine dehydrogenase CapO on residue Tyr89, thus triggering its activity [10], [11]. Collectively, BY-kinase autophosphorylation and ability to phosphorylate polysaccharide-synthesizing enzymes represent a key regulatory tool of capsular polysaccharide synthesis and export.

In proteobacteria, BY-kinases are transmembrane proteins with a N-terminal Wzz-like periplasmic domain and two transmembrane helices [12]. Low-resolution electron microscopy analysis of the BY-kinase Wzc from *E. coli* [13] showed that it forms a bell-shaped structure similar to the polysaccharide co-polymerase Wzz [14] (PDB ID 3B8O). The high-resolution crystal structure of the cytoplasmic kinase domain of the non-phosphorylated form of Wzc [15] (PDB ID 3LA6) demonstrated that it forms an octameric ring. In this organization, the Y-cluster of each subunit is bound to the active site of the neighboring subunit for trans-autophosphorylation. On the other hand, the crystal structure of the cytoplasmic kinase domain of the phosphorylated form of the Wzc orthologue Etk from *E. coli* [16] (PDB ID 3CIO) showed that it forms a monomer with a flexible and unstructured Y-cluster. Altogether, it is proposed that cyclic autophosphorylation and dephosphorylation of BY-kinases regulates association/dissociation of the octamer and thus the functioning of the capsule assembly machinery [4].

**Figure 1:**
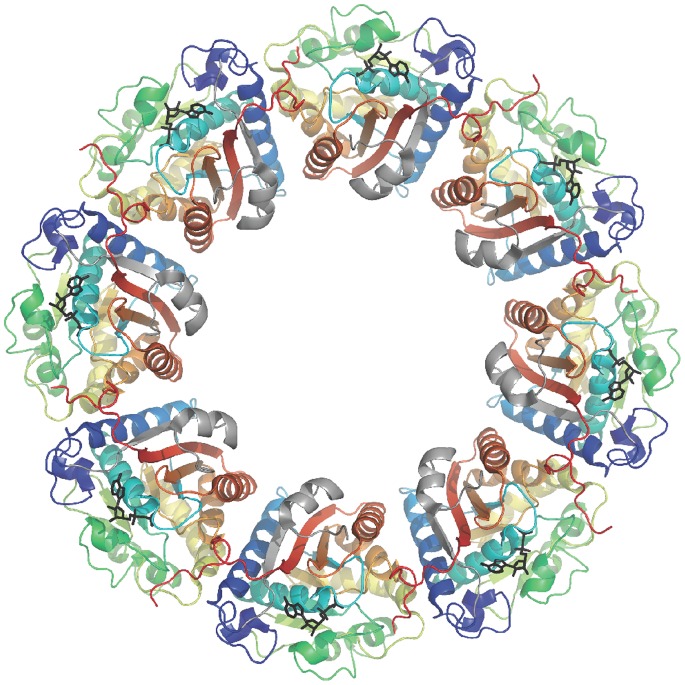
The CapA1B2 octameric ring. The octameric structure of the non-phosphorylated CapA1B2(K55M) mutant protein in complex with ADP-Mg [16] (PDB ID 2VED) is represented as cartoon. In each subunit, the αA-βA elements from CapA1 are colored in grey and the CapB2 moiety is colored by spectrum from blue to red. The bound nucleotides are highlighted as black sticks.

**Figure 2:**
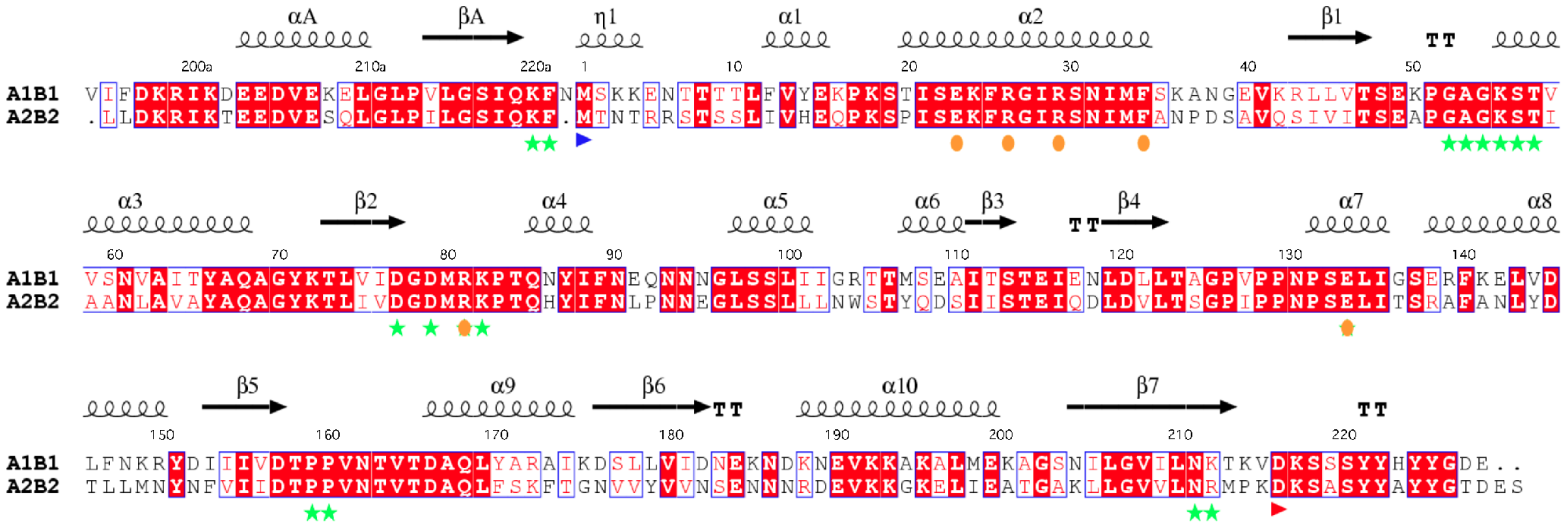
CapA1B1/CapA2B2 sequence comparison. The sequences of the chimera CapA1B1 and CapA2B2 have been aligned using ClustalW [32]. The conserved secondary structure elements as well as the 3_10_-helix η1 only observed in the CapA1B2 structure (PDB IDs 2VED and 3BFV) are indicated at the top of the alignment. using Esprit [33]. Residue numbering of CapA1B1 is indicated with CapA1 residues numbered from V194a to N222a and CapB1 residues numbered from M1 to E228. The N-terminal methionine of CapB1 and CapB2 is highlighted by a blue arrow. The active site residues involved in nucleotide binding (including the Walker A motif and the catalytic lysine K55) are highlighted by green stars. The conserved interface residues including the conserved EX_2_RX_2_R motif in helix α2 are highlighted by orange dots. The first residue of the Y-cluster is indicated by a red arrow.

In firmicutes, the situation is quite similar except that BY-kinases are split into two proteins: one corresponds to the cytoplasmic catalytic domain of proteobacterial BY-kinases (about 230 residues) and the other is a transmembrane domain homologous to the Wzz-like domain (about 230 residues) of proteobacterial BY-kinases. Interestingly, interaction between these two proteins is required for full tyrosine-kinase activity, thus mimicking the proteobacterial organization. For this reason, the membrane protein is also called transmembrane modulator [8]. The C-terminal juxtamembrane domain (last 30 to 50 residues) of the transmembrane modulator has been shown to be essential for triggering the tyrosine-kinase activity of the cognate cytoplasmic protein [17]. More precisely, the structural characterization of the BY-kinase CapB2 from *S. aureus* fused to the juxtamembrane domain of the transmembrane modulator CapA1 [18] (PDB IDs 2VED & 3BFV) showed that the CapA1B2 chimera forms an octameric ring similar to the cytoplasmic domain of Wzc (Figure 1) and that the C-terminal extremity of CapA1 is similar to the juxtamembrane region of Wzc, forming a αA-βA motif (Figure 2). This structure-function analysis demonstrated that the penultimate phenylalanine residue of CapA1 is directly involved in nucleotide binding, thus explaining the activation mechanism. It also suggested that, in firmicutes, trans-autophosphorylation of the C-terminal Y-cluster could induce the dissociation of the octamer, eventually resulting in the release of monomeric cytoplasmic kinase domains from the octameric transmembrane modulator.

In *Staphylococcus aureus*, two BY-kinases (CapB1 and CapB2) together with their cognate transmembrane modulators (CapA1 and CapA2) have been identified [17]. CapA1 and CapB1 are part of the *cap* operon coding for proteins involved in the biosynthesis of capsular polysaccharides while CapA2 and CapB2 are located elsewhere in the genome. Although both BY-kinases share more than 70% sequence similarity (Figure 2), only CapB2 displays an autophosphorylation activity *in vitro*. Even more surprisingly, despite the high sequence similarity (72% identity over the 30 residues of the juxtamembrane fragments) and the conservation of either a terminal (CapA2) or penultimate (CapA1) phenylalanine residue involved in nucleotide binding (Figure 2), CapA1 has been shown to activate CapB2 more efficiently than its cognate modulator CapA2 [17].

To understand such divergent observations between the two homologous proteins, we solved the crystal structures of both CapA1B1 and CapA2B2 chimera and compared them with the structure of CapA1B2 [18]. Although the three structures are very similar, we pointed out small structural differences, which might explain the kinase-dead activity of CapB1. The relevance of these hypotheses was tested by *in vitro* biochemical experiments using site directed mutant proteins. However, none of our observations could explain the null kinase activity of CapB1. We tried to find out if CapB1 could hydrolyze ATP or transfer the γ-phosphate of the bound ATP to another protein substrate. However no evidence of such activity could be found in our assays. We thus discuss if the defective kinase activity of CapB1 observed *in vitro* corresponds to an authentic difference between CapB1 and CapB2 serving as yet uncharacterized purpose in *S. aureus* or reveals that other cellular partners are required for full tyrosine-kinase activity of CapB1 *in vivo*.

## Results and Discussion

### Why is CapA2 a Less Efficient Activator than CapA1?

Preliminary characterization of CapB2 [17] demonstrated that its tyrosine-kinase function is less efficiently activated by its cognate transmembrane modulator CapA2 than by CapA1, which gene is located somewhere else on the genome. In order to find out the structural basis of this discrepancy, we solved the crystal structure of the chimera CapA2B2 (last 27 C-terminal amino acids of CapA2 fused to the N-terminus of CapB2) [17].

**Figure 3:**
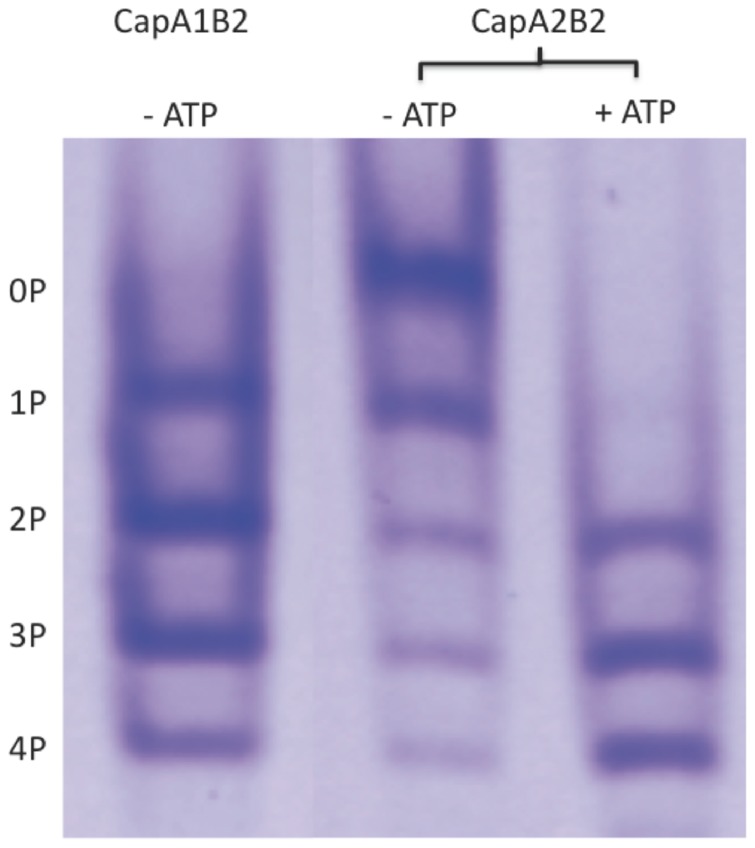
CapA2B2 phosphorylation state analysis. Electrophoretic profile of CapA1B2 and CapA2B2 directly after purification (− ATP) or after 4h incubation at 37°C with 200 µM ATP-Mg (+ATP). The proteins were loaded on a 12.5% non-denaturing polyacrylamide gel and stained with Coomassie Brilliant Blue. The five observed bands were labeled according to the mass spectrometry analysis previously performed on CapA1B2 [16]: the upper band corresponds to the non-phosphorylated form of the protein (0P), and the lower band to the fully phosphorylated form (4P).

Non-denaturing gel electrophoresis demonstrated that the purified CapA2B2 sample displayed the five bands (Figure 3) characteristic of the heterogeneously phosphorylated C-terminal Y-cluster of CapB2. Previous mass spectrometry analysis demonstrated indeed [18] that each band corresponds to a different phosphorylation level, from 0 to 4 phosphorylated residues among the 4 tyrosines 221, 222, 224 and 225 of the Y-cluster (Figure 2). This result demonstrated that the Y-cluster of the CapA2B2 chimera from *S. aureus* autophosphorylated spontaneously during its production process in *E. coli* cells. Autophosphorylation was however less efficient than for CapA1B2 since addition of ATP was required to observe the fully phosphorylated form of CapA2B2 (Figure 3).

**Figure 4:**
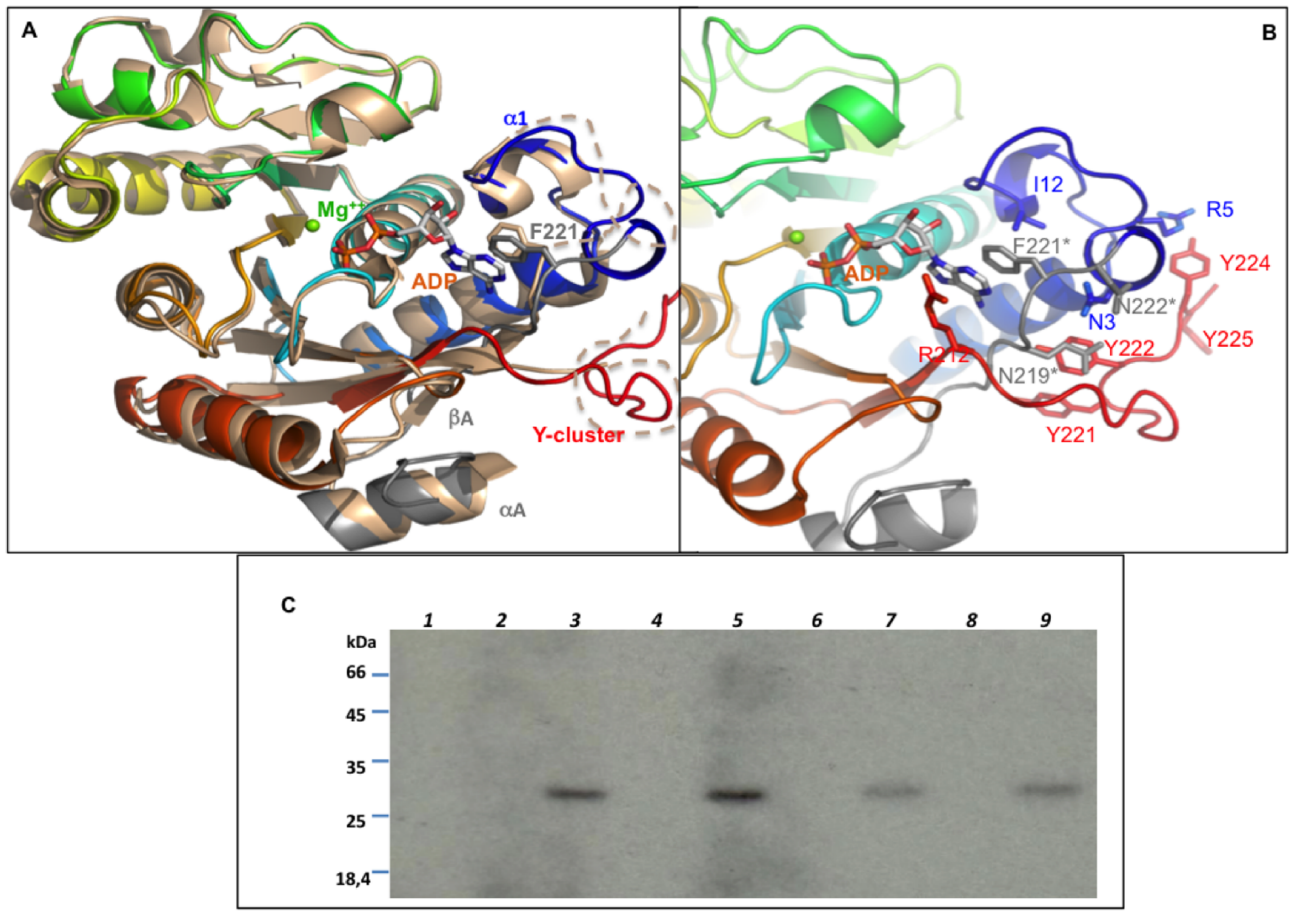
Comparison CapA1B2/CapA2B2. A – Superimposition of the CapA2B2 structure on a monomer from the CapA1B2(K55M)/ADP-Mg octamer (PDB ID 2VED). The proteins are shown as cartoon. CapA1B2(K55M) is colored by spectrum from blue to red. The CapA1 region is highlighted in grey and labeled (αA, βA). The CapA2B2 structure is colored in wheat. The disordered Y-cluster and loop βA-α1 are highlighted as dashed lines. Residues F221* from CapA1 and F220 from CapA2 are shown in sticks as well as the ADP molecule bound in the CapA1B2(K55M) structure. **B** – Interaction networks of the CapA1 C-terminus and of the CapB2 N-terminus. Close view of the CapA1B2(K55M) structure shown in cartoon colored by spectrum. The bound ADP is shown in sticks with the Mg^2+^ ion as a green sphere. Residues discussed in the text are highlighted in sticks. CapA1 residues are labeled with a star. **C** – Effect of CapA1 residue Asn222 on CapB2 activity. CapB2 was incubated in the presence of radioactive ATP and different (wild type or mutated) CapA1 or CapA2 cytoplasmic C-terminal end peptides (CapA1Ct and CapA2Ct). CapB2 autophosphorylation was then analyzed by SDS-PAGE and autoradiography. (lane 1) CapB2; (lane 2) CapA1Ct; (lane 3) CapB2 and CapA1Ct; (lane 4) CapA1Ct deleted from Asn222 (CapA1CtΔN222); (lane 5) CapB2 and CapA1CtΔN222; (lane 6) CapA2Ct; (lane 7) CapB2 and CapA2Ct; (lane 8) CapA2Ct with a C-terminal additional Asn221 (CapA2Ct+N221); (lane 9) CapB2 and CapA2Ct+N221.

The crystal structure of the heterogeneously phosphorylated sample of CapA2B2 crystallized in the absence of nucleotide displays an rmsd of 0.8 Å over 225 Cα atoms aligned with the CapA1B2/ADP-Mg model (PDB code 3BFV) used for phasing. As previously observed in the CapA1B2/ADP-Mg complex [18], the phosphorylated C-terminal Y-cluster of CapB2 is disordered and analysis of the crystal contacts using PISA [19] confirmed that the protein is a monomer. The CapA2 N-terminal part of the protein adopts the same αA-βA conformation than that of the CapA1 fragment. Interestingly, CapB2 residues 1 to 8 linking βA to helix α1 are disordered (Figure 4A). This could be due to the absence of bound nucleotide. Unlike the CapA1B2(K55M) structure that contained bound ADP-Mg while no nucleotide was added to the crystallization solution [18], the active site of CapA2B2 did not reveal any electron density corresponding to a nucleotide that would have co-purified from *E. coli.* This is in agreement with a previous study showing that CapA2B2 displays a weaker affinity for nucleotides compared to CapA1B2, with respective Kd values for Mant-ATP of 190 nM and 50 nM [17]. The discrepancy between the CapA1 and CapA2 activation efficiencies has also been observed when using wild type CapB2 and a GST fusion of the juxtamembrane domain of the modulators. Indeed, it was showed that GST-CapA1 is a better activator than GST-CapA2 [17], demonstrating that the reduced efficiency of CapA2 is not an artifact due to the chimeric bond but seems rather to be linked to its reduced capacity to increase the affinity of CapB2 for nucleotide.

### Investigating the Role of the CapA1 Residue Asn222

The penultimate residue Phe221 of CapA1 has been shown to be directly involved in ATP binding *via* a strong hydrophobic interaction with the base part of the nucleotide [18]. This essential residue corresponds to the last C-terminal residue Phe220 of CapA2 (Figure 2). In the apo structure of CapA2B2, Phe220 is slightly shifted compared to the CapA1 Phe221 residue stacked against the bound adenine in the CapA1B2/ADP-Mg complex (Figure 4A).

The reduced activation efficiency of CapA2 could be explained by the absence of the CapA1 last residue Asn222 (Figure 2) that interacts with the N-terminus of CapB2. As observed in the CapA1B2/ADP-Mg structure, Asn222 is in stacking interaction with Asn3 and H-bonds with main chain atoms from residues 1 and 2, stabilizing a short 3_10_ helix (η1 in Figure 2) that forms a basic platform for the binding of the phosphorylatable Y-cluster, in particular through the Arg5/Tyr224 interaction (Figure 4B). CapB2 N-terminus also forms a hydrophobic pocket allowing proper positioning of the essential phenylalanine residue Phe221, in particular through the Phe221-Ile12 stacking interaction (Figure 4B).

In order to verify the importance of the CapA1 residue Asn222, we produced a GST fused CapA1 cytoplasmic C-terminal fragment deleted from residue Asn222 (GST- CapA1Ct(ΔN222)) and compared its activation efficiency with that of the original GST-CapA1Ct fusion protein [17] (Figure 4C, lanes 3 and 5). Unfortunately, the Asn222 deletion did not affect the CapA1 ability to trigger CapB2 autophosphorylation. Furthermore, we showed that adding a C-terminal asparagine to GST-CapA2Ct also failed to increase CapB2 kinase activity (Figure 4C, lanes 7 and 9).

Only 7 out of the 30 residues of the C-terminal juxtamembrane fragments of CapA1 and CapA2 are not conserved (Figure 2). These substitutions concern residues located at the surface of the protein that do not interact with CapB2. Therefore, they cannot explain the CapB2 activation discrepancy observed *in vitro*. However, it is not clear if this discrepancy also exists *in vivo* where another protein could be required for CapA2-dependent CapB2 activation.

The N-terminal ends of CapA1 and CapA2 are also cytoplasmic and their function in BY-kinase activation has never been investigated. Thus, a stricter reliance on CapA2 N-terminal end for CapB2 activation than on CapA1 could be envisaged. However, it cannot be excluded that the lower activation efficiency of CapA2 could also correspond to distinct physiological purposes.

### Why would CapB1 be Inactive?

**Figure 5:**
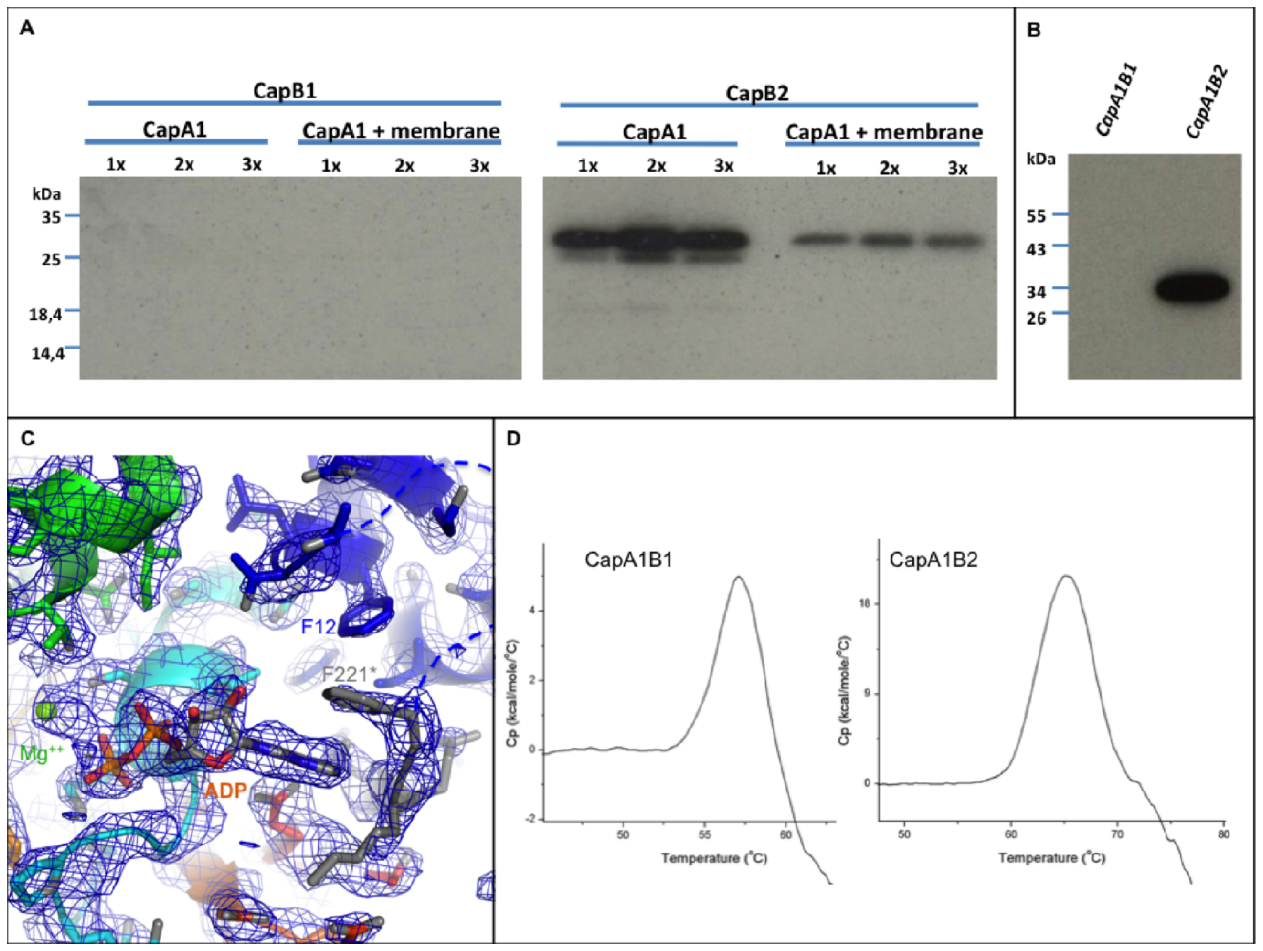
Functional and structural characterization of CapA1B1. A - CapB1 (left panel) and CapB2 (right panel) activation assays using either purified full length 6xHis-CapA1-FL (6xHis-CapA1-FL) or membrane fractions enriched with full-length 6xHis-CapA1-FL. An increasing concentration of CapA1 was used as indicated. CapB1 and CapB2 autophosphorylation was determined after incubation with radioactive ATP, SDS-PAGE separation and autoradiography. **B** - Phosphorylation of CapA1B1 and CapA1B2 during overexpression in *E. coli*. CapA1B1 and CapA1B2 purified from *E. coli* were analyzed by SDS-PAGE and transferred onto a PVDP membrane. Their phosphorylation was revealed by immunoblotting using the anti-phosphotyrosine antibody 4G-10 and the secondary antibody HRP conjugate after direct film exposure. **C** - Electron density of the ADP-Mg molecule bound in the CapA1B1 active site. (2Fo-Fc) map contoured at 1 σ. The bound nucleotide and the residues involved in nucleotide and Mg^2+^ binding are shown in sticks. **D** - Stability of CapA1B1. Differential Scanning Calorimetry of CapA1B1 compared with CapA1B2.

*In vitro* assays did not show any autophosphorylation of CapB1, neither alone or in the presence of CapA1 or CapA2 [17]. We checked if this result was not an experimental artifact due to the use of a cytoplasmic chimera by testing the activity of wild-type CapB1 either in the presence of purified full-length 6xHis-CapA1 (6xHis-CapA1-FL) or in the presence of membrane fractions enriched with full-length 6xHis-CapA1-FL. However, the use of these more biological conditions did not allow us to restore the CapB1 autophosphorylation activity (Figure 5A). We thus analyzed the phosphorylation state of our purified CapA1B1 sample using immunodetection with anti-phosphotyrosine antibodies (Figure 5B). As expected no phosphorylation signal was detected for CapA1B1 in conditions where CapA1B2 is efficiently labeled. Thus, in contrary to CapA1B2, CapA1B1 did not spontaneously autophosphorylate during the production in *E. coli* cells.

According to the regulatory mechanism proposed for the BY-kinases [4], the inactive CapA1B1 protein was thus supposed to form the octameric ring observed for the inactive mutants CapA1B2(K55M) [18] and Wzc_cyto_(K540M) [15]. To test this and further understand why CapB1 is less active than CapB2, we solved the crystal structure of the CapA1B1 chimera. The crystal structure of the CapA1B1/ADP-Mg complex displays an rmsd of 0.9 Å over 229 Cα atoms aligned with the CapA1B2/ADP-Mg model (PDB code 3BFV) [18] used for phasing.

Clear electron density corresponding to a bound ADP molecule and associated Mg^2+^ ion was observed in the active site of CapA1B1 (Figure 5C). Despite the presence of the bound nucleotide, CapB1 residues 1 to 8 as well as the CapA1 last residue Asn222 are disordered. This suggests that the similar disorder observed in the CapA2B2 structure is not due to the absence of nucleotide. The stacking interaction between the CapA1 Phe221 and the base part of the bound ADP is conserved. Phe221 is stabilized in this position by a T-shaped hydrophobic interaction with CapB1 residue Phe12 (Figure 5C) replacing the stacking interaction with CapB2 residue Ile12 observed in the CapA1B2 structure (Figure 4B). Surprisingly, despite the inactivity of the protein, the CapA1B1 structure does not display the expected octameric conformation and the Y-cluster is disordered. This suggests that CapB1 is intrinsically in the monomeric active conformation compatible with the binding of specific protein substrate [18]. This hypothesis is supported by the conservation of the active site residues (Figure 2). Thus, although not autophosphorylating, CapB1 could be able to phosphorylate other endogenous substrates.

The basic isoelectric point of CapB1, with a calculated value of 8.8 (compared with 5.45 for CapB2), suggests that the formation of a CapB1 oligomer compatible with trans-autophosphorylation could be impaired by unfavorable electrostatic surface charges. Unfortunately, this hypothesis could not be verified since the octameric form of the BY-kinases is very difficult to observe in solution, even with the inactive mutants CapA1B2(K55M) [18] and Wzc_cyto_(K540M) [15]. Electron microscopy analysis of the CapA1/CapB1 and CapA2/CapB2 transmembrane complexes would thus be helpful to elucidate their biological organization.

Differential scanning calorimetry measurements performed at pH 7.8 revealed that, in these conditions, CapA1B1 is less stable than CapA1B2, with respective Tm values of 56.8°C and 66.2°C (Figure 5D). We thus evaluated if the basic nature of CapB1 could influence the optimal pH of the *in vitro* assays. However, the use of buffers with pH ranging from 5 to 8 had no effect on CapA1B1 autophosphorylation and the protein remained inactive (data not shown).

### Investigating the Role of the CapB N-terminus

**Figure 6:**
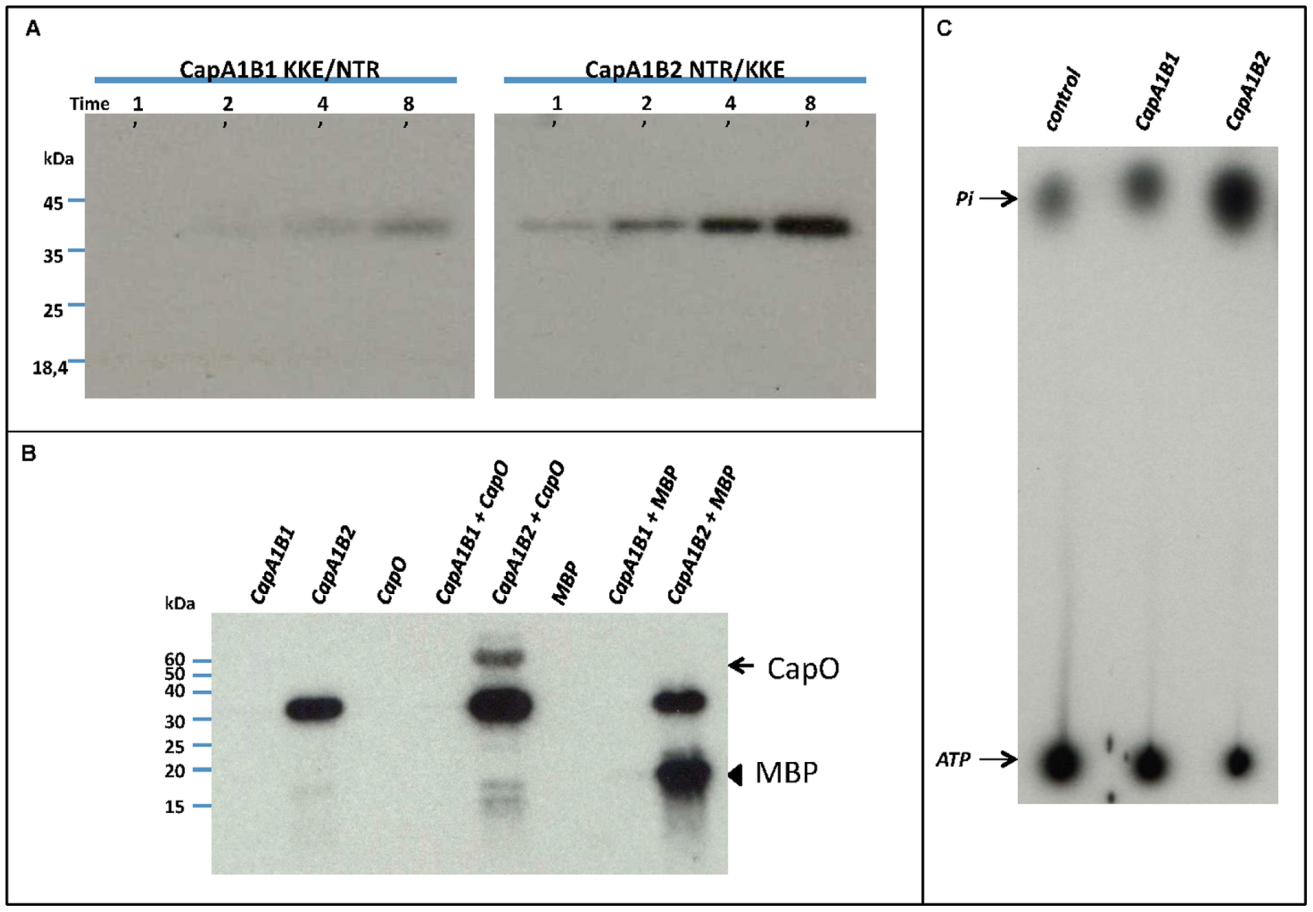
CapA1B1 activity assays. A – Effect of CapB2 N3T4R5 substitution for CapB1 K3K4E5 and vice versa on CaB1 and CapB2 kinase activity. CapA1B1(KKE/NTR) (left panel) and CapA1B2(NTR/KKE) (right panel) were incubated in the presence of radioactive ATP for either 1, 2, 4 or 8 minutes. After SDS-PAGE analysis, their autophosphorylation was visualized by autoradiography. **B** – CapO and myelin-binding protein (MBP) phosphorylation assays. The ability of CapA1B1 and CapA1B2 to phosphorylate CapO or the myelin-binding protein (MBP) was determined after incubation in the presence of radioactive ATP, SDS-PAGE analysis and film exposure. The arrow points to CapO whereas the arrowhead shows the MBP. **C** – ATPase activity of CapA1B1 and CapA1B2. The hydrolysis of [γ-32P] ATP was measured in the presence of either CapA1B1 or CapA1B2 as described under “Materials and Methods”. Migration of a control reaction mixture devoid of CapA1B1 or CapA1B2 is also shown.

We tried to confirm that the lack of autophosphorylation activity of CapB1 could be related to its incapacity to oligomerize. Half of the octamer interface involves the conserved EX_2_RX_2_R motif of helix α2 [3], [17] (Figure 2) forming salt bridges with surface loops from the neighbouring subunit. This conserved contact area has been shown to be essential for both trans-autophosphorylation and polysaccharide synthesis [15]. The other 50% of the interface area is provided by the Y-cluster [18]. This suggests that its proper positioning is essential for the stability of the octamer. The residues involved in the binding of the Y-cluster in CapB2 are conserved in CapB1 except for few N-terminal residues, including the previously mentioned Arg5 that is replaced by a glutamate and Asn3 that is replaced by a lysine (Figure 2). As mentioned above, Arg5 and Asn3 are respectively stacked against Tyr225 of the Y-cluster and Asn222 from CapA1. Asn3 also makes H-bonds with the CapA1 residue Gln219, which is stacked against Tyr222 of the Y-cluster (Figure 4B). The altered N-terminal sequence of CapB1 could thus result in a decreased affinity for the phosphorylatable Y-cluster and a destabilization of the octamer. In order to assess the role of the non-conserved N-terminal residues on the trans-autophosphorylation activity, we exchanged residues N3T4R5 of CapB2 and K3K4E5 residues of CapB1 and tested the activity of the resulting mutant chimera CapA1B1(KKE/NTR) and CapA1B2(NTR/KKE). However, these mutations did not restore the activity of CapB1 nor inhibit CapB2 (Figure 6A), indicating that these N-terminal residues do not play the essential role suggested by the structure analysis.

### Could CapA1B1 Phosphorylate an Endogenous Substrate?

Autophosphorylation allows BY-kinases to shift from an inactive octameric state to a monomeric active conformation compatible with the binding of their specific protein substrates [18]. This mechanism is supported by *in vitro* experiments showing that CapB2 devoid of its Y-cluster is also able to phosphorylate CapO [18]. The apparent incapacity of CapB1 to form an octamer suggests that it could be intrinsically in the active conformation, ready to phosphorylate endogenous substrates. We thus tested the capacity of CapA1B1 to phosphorylate CapO, the UDP-N-acetylmannosamine dehydrogenase of *S. aureus* that has been shown to be phosphorylated by CapB2 on residue Tyr89 [11]. Unfortunately, CapA1B1 was unable to phosphorylate CapO (Figure 6B). Nevertheless, it could not be excluded that the different electrostatic surface displayed by CapA1B1 and CapA1B2 could be responsible for distinct substrate specificity. We then also tested CapB1 ability to phosphorylate the myelin-binding protein (MBP), a protein classically used to detect kinase activity in biological samples. However, once again, while CapA1B2 efficiently phosphorylated the MBP, CapA1B1 did not (Figure 6B).

Finally, we checked whether or not CapA1B1 could hydrolyze ATP. We thus analyzed the ATPase activity of CapA1B1 using radioactive [γ-^32^P]ATP (Figure 6C). However, in conditions where CapA1B2 produced inorganic radioactive phosphate at the expense of ATP, we did not observe any ATP hydrolysis in the presence of CapA1B1. Altogether, this data prove that CapB1 does not possess a tyrosine-kinase activity despite its BY-kinase signature [3] and a 3D structure very similar to that of the active BY-kinase CapB2. Unfortunately, our structural and biochemical analysis failed to provide any explanation for CapB1 inactivity.

## Conclusion and Perspectives

**Figure 7:**
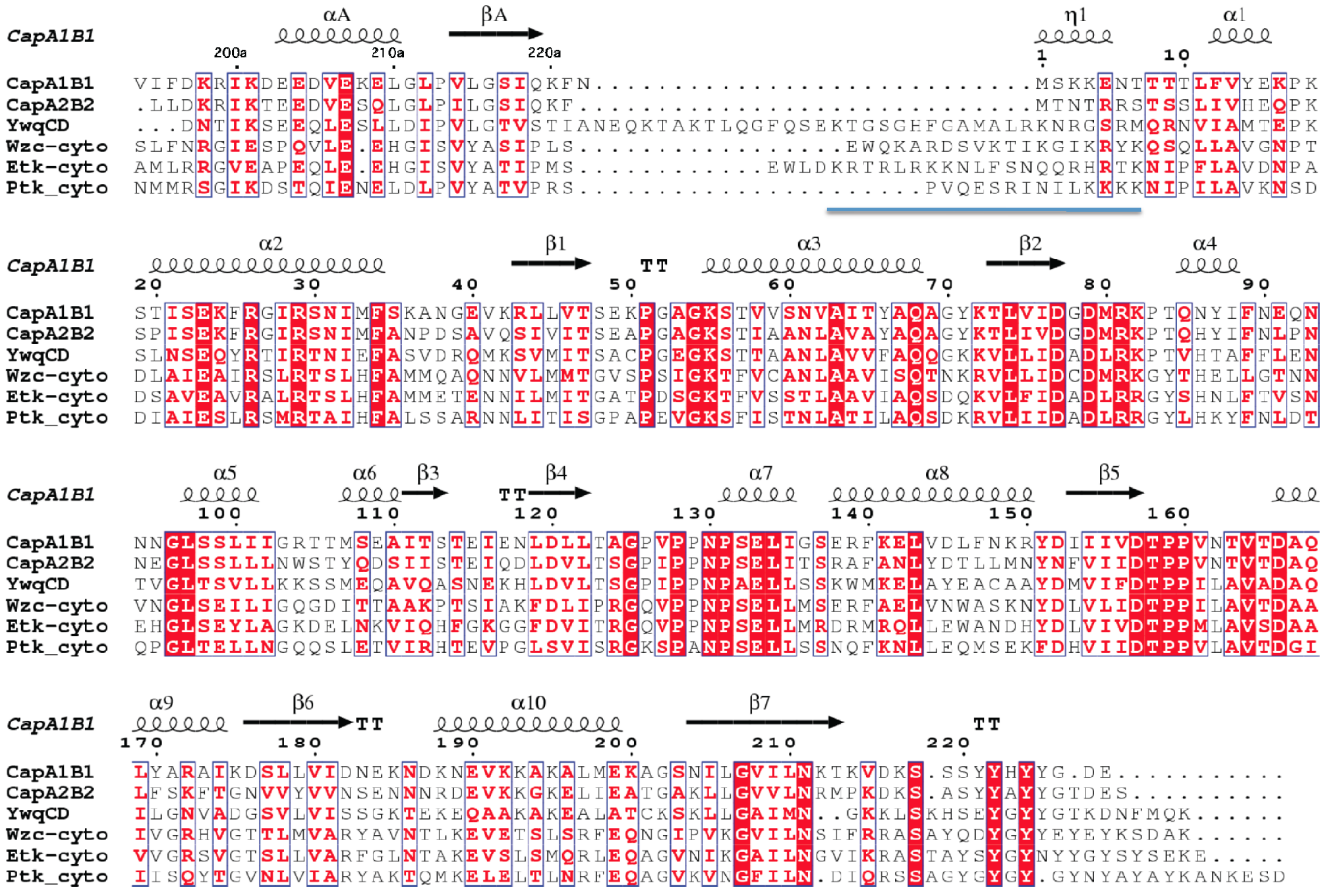
Sequence comparison of BY-kinases from firmicutes and proteobacteria. The sequences from CapA1B1 and CapA2B2 from *S. aureus* have been aligned with the chimera YwqCD constructed with the proteins YwqC and YwqD from *B. subtilis* (Swiss-Prot entries P96715 and P96716, respectively) as well as with the cytoplasmic domain of the proteobacterial BY-kinases Wzc and Etk from *E. coli* and Ptk from *Acinetobacter johnsonii* (Swiss-Prot entry O52788). The CapA1B1 residue numbering and secondary structure elements are shown at the top of the alignment as in Figure 2. The RK-cluster region is underlined.

**Figure 8:**
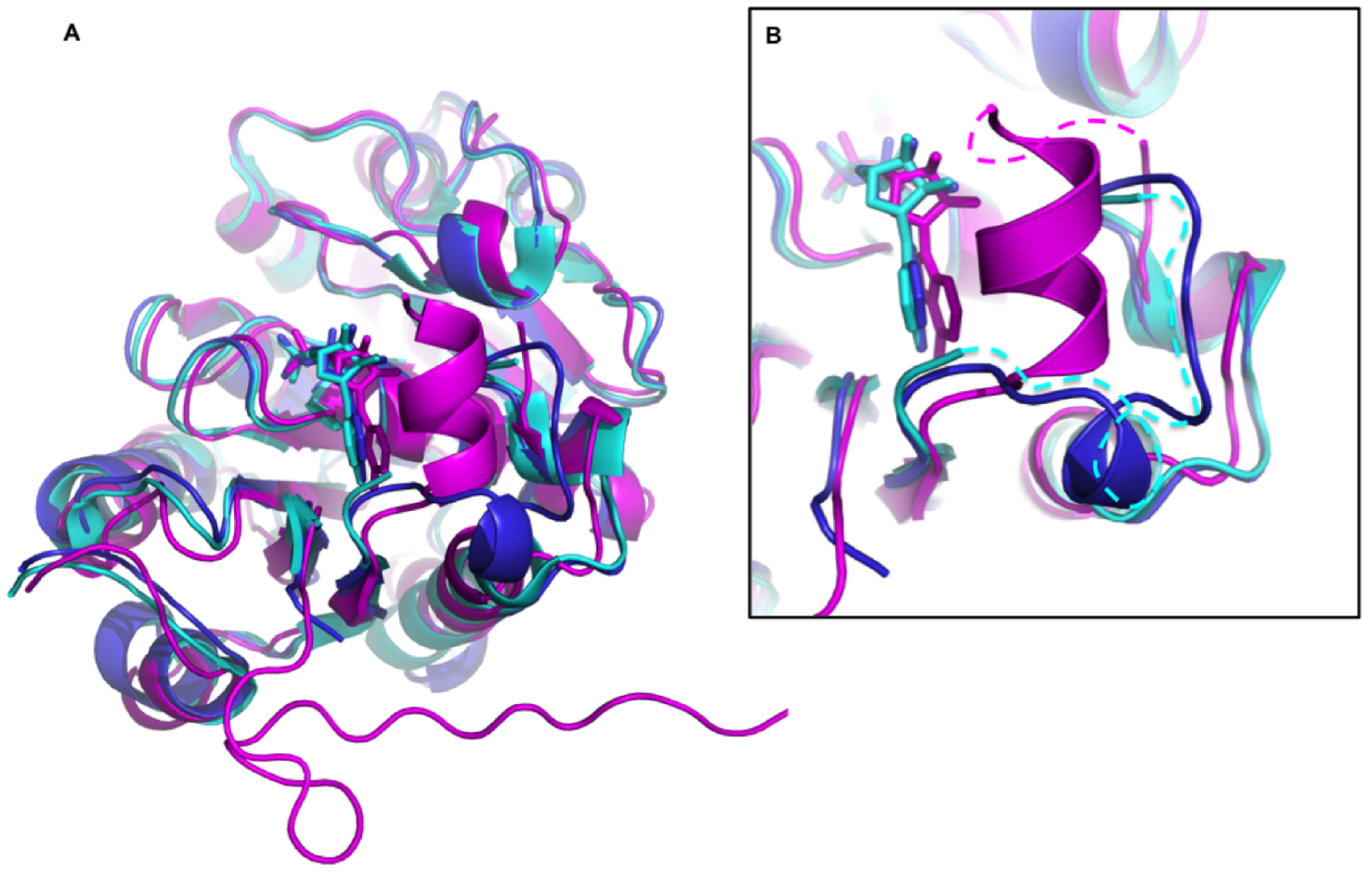
Structural comparison CapAB/Wzc. A – Global superimposition. The structure of CapA1B1 (in cyan) has been superimposed with the CapA1B2 phosphorylated monomer (in blue) (PDB ID 3BFV) as well as with a subunit of the non-phosphorylated Wzc(K540M) octamer (in magenta) (PDB ID 3LA6). The structures are shown in cartoon with bound ADP molecules in sticks. **B** – Close view of the RK-cluster region. The flexible parts of the CapA1B1 and Wzc structures are highlighted as dashed lines.

Although our *in vitro* assays failed to detect any activity for CapB1, this inactivity cannot be explained by our structural analysis. We propose that the poorly conserved N-terminus of CapB1 and CapB2 could be responsible for their different behaviors. An interesting hypothesis concerning the role of this flexible extremity could come from the comparison with the structures of the BY-kinases Wzc [15] and Etk [16] from *Escherichia coli*. The latter display in this region a flexible loop containing a basic insert called RK cluster (Figure 7) that has been shown to be essential for the exopolysaccharide co-polymerase activity of the protein. It has also been proposed to influence Wzc and Etk tyrosine-kinase activity through protein-protein interactions with other components of the capsule synthesis machinery [15]. Interestingly, an even longer basic insert is also observed in the chimera YwqCD from the firmicute *Bacillus subtilis* (Figure 7). Superimposition of the CapAB and Wzc structures (Figure 8) suggests that the CapAB N-terminus could play a similar role to the Wzc RK-cluster in the multiprotein transmembrane complex. These interactions could be essential for CapB1 activity and play a minor role for CapB2.

On the other hand, we cannot completely rule out that CapB1 would be indeed inactive *in vivo*. On this basis, one would also wonder what would be the benefit for *Staphylococcus* to produce an inactive BY-kinase. A possible answer is that CapB1 and CapB2 could fulfill distinct cellular functions. Supporting this idea, evidences have been provided that the splitting into two distinct proteins allows BY-kinases from firmicutes to participate in the regulation of distinct cellular processes [7]. The analysis of strains deficient for *capB1, capB2, capA1* and/or *capA2* should help clarify the respective cellular function of the two systems. Another hypothesis lies in the duplication of BY-kinase genes in several genomes of bacteria [3]. For example, in *B. subtilis*, a similar situation is observed with an active PtkA and an inactive PtkB [8]. One could assume that an inactive BY-kinase could be a regulatory mean to interfere with the cellular function of the active one.

A parallel can be drawn with eukaryotic proteins termed pseudokinases that contain a kinase-like domain but catalytically inactive [20]. Much remains to be learnt about the intricate functions of pseudokinases. However, it is proposed that they could act as molecular scaffolds or allosteric factors to promote the activity of other protein-kinases. To illustrate this, the ILK (Integrin-linked kinase) pseudokinase functions as a scaffolding protein mediating interactions with several proteins via multiple sites including its pseudoactive site and is crucial in cell-extracellular matrix adhesions [21]. On the other hand, the HER3 (also called EGFR or ErbB1) pseudokinase domain allosterically activates the functional tyrosine kinase domain of HER2 (ErB2) and contributes to the development, proliferation and survival of tumor cells [22], [23]. Like for CapB1, attempts to convert human HER3 to an active kinase by reinstating catalytic important residues have failed to restore activity [24]. A very interesting and hypothesis would bet thus that the inactive CapB1 behaves like a pseudokinase and plays an important role for CapB2 to participate in the regulation of some cellular processes including polysaccharide production. This assumption is supported by a recent study reporting that in bacteria some cellular processes could also require interplay between an active protein-kinase and an inactive pseudokinase. Indeed, the protein-kinase PknB of *Mycobacterium tuberculosis* operates as key regulator of peptidoglycan biosynthesis through its interaction with the pseudokinase domain of MviN [25].

Answering all these questions is challenging and will required comprehensive cellular analysis. Complicated as it may be, those future studies should lead to our better understanding of BY-kinases mediated regulatory functions of the bacterial cell physiology.

## Materials and Methods

### Cloning and Site Directed Mutagenesis

*Escherichia coli* XL1-Blue cells were used as a host strain for DNA manipulations. Protein overexpression was performed using *E. coli* BL21(DE3) strain. Luria–Bertani (LB) broth was used for growth and expression, supplemented when required with tetracycline and/or ampicillin to a final concentration of 15 µg/ml and 100 µg/ml, respectively. Details concerning bacterial strains and plasmids used in this study are summarized in Table 1. Site-directed mutagenesis was carried out by using polymerase chain reaction and primers described in Table 1. Briefly, plasmids pQE30-Cap5A1B2 and pQE30-Cap5A1B1 were used as templates in order to introduce the triple substitutions N3T4R5 for K3K4E5 and K3K4E5 for N3T4R5 in CapA1B2 and CapA1B1, respectively. The mutated and amplified DNA fragments were digested with either *Bam*H1 and *Hind*III or *Bam*HI and *Pst*I, for CapA1B2 and CapA1B1, respectively, and ligated with pQE30 vector previously linearized with the same restriction enzymes. Asn222 deletion in CapA1Ct or insertion in CapA2Ct were achieved using respectively plasmids pGEXVM-A1Ct and pGEXVM-A2Ct as templates and primer pairs A1ctΔN222 (−)/A1ct (+) and A2ct+N222 (−)/A2ct (+), respectively. The amplified DNA fragments were digested using *Bam*H1 and *Hind*III and introduced into the pGEXVM vector previously linearized with the same restriction enzymes.

**Table 1:** Bacterial strains, plasmids and primers used in this study.

Strain	Description	Reference
XL1-Blue	*sup*E44 *hsd*R17 *rec*A1 *end*A1 *gyr*A46 *thi rel*A1 *lac^−^,*[*pro*AB^+^ *lac*I^q^ *lac*ZΔM15 *Tn*10 (Tet^R^)]	Bullock *et al.*, 1987
BL21 pRep4-GroESL	*F′, dmc, ompT, hsdS (r^−^_B_m^−^_B_) galλ, pRep4-groESL*	Amrein *et al.*, 1995
**Plasmid**	**Description**	**Reference**
pQE30	Expression vector generating His_6_ fusion protein, Amp^R^	Qiagen
pGEXVM	Expression vector generating GST fusion protein, Amp^R^	Molle *et al.*, 2003
pQE30-Cap5A1B1	Encoding last 29 amino acids of Cap5A1 (from Val194 to Asn222) fused to Cap5B1 (from Met1to Glu228), His_6_A1CtB1, cloned in BamHI/PstI sites, Amp^R^	Soulat *et al.*, 2006
pQE30-Cap5A1B2	Encoding last 29 amino acids of Cap5A1 (from Val194 to Asn222) fused to Cap5B2 (Met1 toSer230), His_6_A1CtB2, cloned in BamHI/HindIII sites, Amp^R^	Soulat *et al.*, 2006
pQE30-Cap5O	Encoding Cap5O, His_6_CapO (from Met2 to Lys437), cloned in BamHI/PstI sites, Amp^R^	Soulat *et al.*, 2007
pQE30-Cap5A1B1 KKE/NTR	Same as pQE30-Cap5A1B1 but mutated on K3N,K4T and E5R	This study
pQE30-Cap5A1B2 NTR/KKE	Same as pQE30-Cap5A1B2 but mutated on N3K,T4K and R5E	This study
pGEXVM-A1Ct	Encoding last 29 amino acids of Cap5A1 from Val194 to Asn222, GSTA1Ct, cloned inBamHI/HindIII sites, Amp^R^	Soulat *et al.*, 2006
pGEXVM-A1CtΔN222	Same as pGEXVM-A1Ct but deleted of Asn222	This study
pGEXVM-A2Ct	Encoding last 27 amino acids of Cap5A2 from Leu194 to Phe220, GSTA2Ct, cloned inBamHI/HindIII sites, Amp^R^	Soulat *et al.*, 2006
pGEXVM-A2Ct+N222	Same as pGEXVM-A2Ct but with a additional C-terminal Asn	This study
pET15b-A1-FL	Encoding Cap5A1 from Met1 to Asn222, His_6_A1, cloned in NdeI/BamHI sites, Amp^R^	Soulat *et al.*, 2006
pET15b-B1	Encoding Cap5B1 from Met1 to Glu228, His_6_B1, cloned in NdeI/BamHI sites, Amp^R^	Soulat *et al.*, 2006
pET15b-B2	Encoding Cap5B1 from Met1 to Ser230, His_6_B2, cloned in XhoI/BamHI sites, Amp^R^	Soulat *et al.*, 2006
**Primer**	**5**′ **to 3**′ **sequence** a,b,c	**Reference**
B1 (−)	TGCA*CTGCAG*TTATTCATCTCCATAATAGTGAT	This study
B2 (−)	CCC*AAGCTT*TCATGATTCATCAGTCCCATAATA	This study
A1ct (+)	TAT*GGATCC*AAAGTAATTTTCGATAAGCG	This study
A2ct (+)	TAT*GGATCC*TTATTAGATAAGCGTATTAAGACAG	This study
A1B2 N3KT4K R5E (−)	CTTGATGTACTTCTTCTT**TC**T**T**T**T**TTCGTCATATTAAATTTTTG	This study
A1B1 K3NK4T E5R (−)	GTGTTGTTGTCGTATTT**CG**C**G**T**A**TTTGACATATTAAATTTTTG	This study
A1ctΔN222 (−)	TAT*AAGCTT*TTAAAATTTTTGAATTGAACCCAATAC	This study
A2ct+N222 (−)	TAT*AAGCTT*TTAATTAAATTTTTGTATTGAACCTAAAATAGG	This study

a Forward and reverse primers are represented by plus (+) or minus (−), respectively.

b restriction sites are italicized.

c The bases mutated from those present in the wild type are bold.

### Protein Expression and Purification

The wild type as well as mutated 6xHis-tagged proteins were overexpressed and purified as described previously [10]. Briefly, the proteins were expressed in *E. coli* BL21(DE3) strain at 28°C. They were purified at 4°C by IMAC immediately followed by gel filtration on a Superdex S75 column. The purified samples were concentrated using Centricon centrifugal filter units (Millipore) of 10 kDa molecular weight cut-off and stored at –20°C in a buffer containing 25 mM Bis-Tris, pH 6.5, 150 mM NaCl, and 10% glycerol. GST-fused CapA1 and CapA2 proteins were purified as previously described [15].

### Crystallization

Crystallization conditions for the CapA1B1 and CapA2B2 proteins were determined at 18°C using commercial crystallization screening kits and sitting drop method. Liquid handling was performed by a nanodrop crystallization robot (Cartesian). The crystals were optimized manually with homemade solutions using the hanging drop method. CapA2B2 crystallized at 7 mg/ml in 25% PEG 550 MME, 100 mM Hepes, pH 7.5, 5 mM MgCl_2_ in the absence of nucleotide. The best crystals of CapA1B1 were obtained at 4 mg/ml in the presence of 5 mM ADP-Mg in 28% PEG 1000 and 100 mM Tris-HCl, pH 8.5.

### Structure Determination

Crystals were, soaked in a cryoprotectant solution consisting of the reservoir solution supplemented with increasing glycerol concentrations up to 25% (w/v) and subsequently flash-frozen in liquid nitrogen. Diffraction data were collected at the European Synchrotron Radiation Facility (ESRF, Grenoble, France) on beamlines ID23-2 and ID29 for CapA2B2 and CapA1B1, respectively. The CapA2B2 crystals diffracted up to 1.3 Å resolution in space group P2_1_ with 1 molecule per asymmetric unit. The CapA1B1 crystals diffracted up to 2.4 Å resolution in space group P2_1_2_1_2_1_, with 1 molecule per asymmetric unit.

Diffraction data were processed using the program Mosflm [26] and the CCP4i package [27]. Phasing was performed by molecular replacement with the program Phaser [28] using the CapA1B2 structure (PDB code 3BFV) [18] as starting model. Structure refinement was performed using the programs Buster [29] and Phenix [30]. Models were manually optimized using the model-building software COOT [31]. A summary of the refinement and data statistics is given in Table 2.

**Table 2:** Structural data.

Data set	CapA1B1	CapA2B2
**Data collection**		
Space group	P2_1_2_1_2_1_	P2_1_
a, b, c (Å)	41.03, 64.63, 88.28	36.33, 88.67, 39.41
α, β, γ (°)	90.0, 90.0, 90.0	90.0, 115.35, 90.0
Resolution (Å)^a^	50.0–2.2 (2.33–2.20)	35.6–1.3 (1.37–1.30)
Robserved (%)^b^	9.3 (111.3)	8.2 (55.1)
Observed reflections	59 025 (9 419)	143 663 (20 588)
Unique reflections	12 422 (1 958)	54 294 (7 988)
Mean((I)/σ(I))	14.74 (1.93)	9.8 (2.1)
Completeness (%)	99.7 (99.5)	98.3 (99.6)
**Refinement statistics**		
Resolution range used (Å)	44.1–2.2 (2.42–2.20)	33.0–1.3 (1.32–1.30)
Number of used reflections	12 421	54 245
Test set size (%)	5.0	5.0
R_work_ (%)^c^	19.72 (26.87)	17.16 (25.05)
R_free_ (%)	24.20 (32.71)	19.07 (24.82)
Number of protein atoms/a. u.	1774	1769
Number of water molecules	27	207
Ligand	ADP-Mg	none
Isotropic B value (Å^2^)		
- mean	57.56	15.43
- minimum	35.93	5.20
- maximum	91.71	58.68
Rms deviation from ideal values:		
- bond lengths (Å)	0.008	0.006
- bond angles (°)	1.212	1.012
- chirality angles (°)	0.073	0.069
- dihedral angles (°)	15.617	11.055
- planarity (°)	0.005	0.005
Ramachandran statistics (%)		
- favoured	97.7	99.6
- outliers	0.0	0.0

a Numbers in parentheses represent values in the highest resolution shell.

b R_observed_ = Σ_h_Σ_i_ |I(h,i)−<I(h)>|/Σ_h_Σ_i_ I(h,i) where I(h,i) is the intensity value of the i-th measurement of h and <I(h)> is the corresponding mean value of I(h) for all i measurements.

c R_work_ = Σ ||Fobs|−|Fcalc||/Σ |Fobs|, where |Fobs| and |Fcalc| are the observed and calculated structure factor amplitudes respectively. _Rfree_ is the same as R_work_ but calculated with a subset of all reflections (test set) that was never used in crystallographic refinement.

### Kinase and ATP Hydrolysis Assays

*In vitro* phosphorylation of 0.1 µg of different purified proteins was carried out in a reaction mixture containing 25 mM Tris-HCl, pH 7.5, 1 mM DTT, 5 mM MgCl_2_, 1 mM EDTA, and 10 µM ATP with 200 µCi/ml [γ-^32^P]ATP. When appropriated, 1 µg of CapO or 0.5 µg of Myelin Basic Protein (MBP) were added as substrate. After 20 min incubation at 37°C the samples were analyzed using SDS-PAGE. After migration the gels were soaked in 16% TCA for 10 min at 90°C and stained with Coomassie Brilliant Blue. Radioactive proteins were visualized by autoradiography using direct exposure to films.

For ATPase assays, we used the same procedure as previously described [25]. Briefly, proteins were incubated in the presence of radioactive ATP at 5 µM concentration in the same buffer as used in the kinase assays. The reaction was stopped by cooling the reaction mixture at 4°C. One microliter of each sample was analyzed by thin layer chromatography using PEI-cellulose F plates from Merck. The plates were subsequently dried out and analyzed by autoradiography.

### Immnunoblot Analysis

CapA1B1 and CapA1B2 purified samples were loaded on SDS-PAGE and tyrosine autophosphorylation was determined by immunoblotting using the anti-phosphotyrosine polyclonal 4G-10 antibody (Millipore) at 1/2000 dilution. A mouse anti-rabbit secondary antibody HRP conjugate (Biorad) was used at 1/5000 dilution. Membranes were developed using the chemiluminescent substrate Supersignal® West Pico from Thermo.

### Differential Scanning Calorimetry

DSC experiments were performed using a VP-DSC calorimeter (Microcal®). CapA1B1 and CapA1B2 samples were used at a concentration of 10 µM in Tris-HCl 25 mM, pH 7.8, with 250 mM and 100 mM NaCl, respectively. Two successive scans from 20°C to 80°C at a heating speed of 60°C/h were performed for each experiment. Data were analyzed using the MicroCal Origin software provided by the manufacturer.
